# PP37 SUSTAIN-HTA. Structured Collaboration Between Health Technology Assessment Bodies And Academia: Current Practices, Barriers, And Recommendations In Europe

**DOI:** 10.1017/S0266462325102560

**Published:** 2025-12-29

**Authors:** Enric Rubiella, Jessica Ruiz-Baena, Roland Pastells-Peiró, Rosa Maria Vivanco-Hidalgo, Liza van Mun, Carlo Federici, Alessandra Alessandra

## Abstract

**Introduction:**

Health technology assessment (HTA) uses explicit methods to determine the value of a health technology at different stages of its life cycle. Collaboration between HTA bodies and academia is crucial for advancing methodologies and their use in practice. However, regulatory misalignment and resource constraints hinder effective partnerships. This study provided ways to facilitate structured dialogue between HTA bodies and academia.

**Methods:**

A two-phase approach was used. Phase one involved a rapid literature review and snowballing to identify frameworks for HTA–academia collaborations. Phase two consisted of an online survey with targeted dissemination to HTA bodies across Europe and beyond through social media (35 were invited). The survey questions were informed by a rapid review and addressed collaboration types, incentives, challenges, regulatory frameworks, and HTA regulation impact. Key areas included methodological development, training, and structured partnerships. Data were analyzed in Excel using descriptive statistics to identify patterns not only across Europe, but also within distinct European areas. Results were categorized by Europe as a whole and individual countries.

**Results:**

We obtained responses from 17 HTA bodies of which 16 maintained academic collaborations, primarily with universities at a national level. Common structures included service contracts (n=11) and consulting agreements (n=9), enabling short-term flexibility. Current collaborations focused on assessing scientific data (n=12) and specific health technologies (n=11). Barriers included diverse research paradigms (n=5), organizational resistance to change (n=5), and liability concerns (n=5). Respondents emphasized the need for guidelines and capacity building initiatives to overcome these challenges. Methodological innovation (n=14) and capacity building and training (n=13) emerged as priority objectives for collaboration. Future collaborations are expected (n=9) to align with the European HTA Regulation.

**Conclusions:**

Strengthening HTA–academia collaborations requires structured and sustainable infrastructures addressing regulatory barriers, training needs, and cultural differences across countries. Clear guidelines, strategic academic partnership selection, and incentives can enhance collaboration. Implementing these findings will contribute to improved and updated methodologies and evidence-based decision-making in HTA across Europe and beyond.

